# Development of Short Jute Fiber-Reinforced Thermoplastic Pre-Preg Tapes

**DOI:** 10.3390/polym17030388

**Published:** 2025-01-31

**Authors:** Mengyuan Dun, Haitao Fu, Jianxiu Hao, Weihong Wang

**Affiliations:** 1 Key Laboratory of Spin Electron and Nanomaterials of Anhui Higher Education Institutes, School of Chemistry and Chemical Engineering, Suzhou University, Suzhou 234000, China; dunmengyuan0808@ahszu.edu.cn (M.D.); haojx2021@ahszu.edu.cn (J.H.); 2 Key Laboratory of Bio-Based Material Science and Technology (Ministry of Education), Northeast Forestry University, 6 Hexing Road, Harbin 150040, China

**Keywords:** natural fibers, thermoplastic based pre-preg processing, filament winding, impregnation

## Abstract

Jute fibers are renewable, light, and strong, allowing them to be considered as attractive materials in composite manufacturing. In the present work, a simple and effective method for preparing continuous pre-preg tapes from short jute fiber bundles (without twist) is developed and its application in winding forming is evaluated. Linear low-density polyethylene film (LLDPE) with good flexibility and weather resistance was used as the thermoplastic matrix; jute fiber bundles were first spread parallel to each other on an LLDPE film and then rolled up to form a pre-roll. The pre-roll enclosing fiber bundles was hot-pressed in a designed mold to form a pre-preg tape, where the fiber bundles were more parallel to the tape than the fibers in twine. Although the untwisted structure exhibited a lower tensile strength for the fiber bundle, it could be processed into a continuous pre-preg with higher tensile strength than the jute twine-impregnated pre-preg. This is based on the good impregnation of the short fiber bundle and its unidirectional, uniform strengthening in the continuous pre-preg. The tensile strength and modulus of the fiber bundle-reinforced pre-preg increased by 16.70% and 257.14%, respectively, compared with jute twine-reinforced pre-preg (within the fiber proportion of 40.wt%). When applied to winding, the fiber bundle-reinforced pre-preg showed advantages of interlayer fusion, surface flatness, and ring stiffness. In contrast, the twisted continuous structure did not retain its advantage in pre-preg. The development of pre-preg tapes by discontinuous fibers might be a good way for utilizing natural fibers in the field of green engineering due to its diverse secondary processing.

## 1. Introduction

A pre-preg can be defined as a semi-finished product that is pre-impregnated with resin on a continuous fiber or fabric and then processed and stored for later use [1]. Pre-pregs have found important applications in industries such as wind energy, automobiles, sports equipment, pipes, high-pressure vessels, and other industrial equipment [2]. At present, research on thermoplastic pre-pregs is a hot topic compared to thermosetting pre-pregs. This is determined by the advantageous properties of the thermoplastic resin itself (such as good environmental adaptability, short processing cycles, recyclability, strong fracture toughness, high damage tolerance, etc.) [3].

In the preparation of thermoplastic-based pre-pregs, synthetic fibers such as fiberglass, carbon fiber, and polymers fiber are commonly used due to the high level of reinforcement. However, certain drawbacks, such as machine wear, health hazards, huge energy consumption, and serious pollution in the processing and utilization lead to the synthetic fibers not being advocated. Natural fibers derived from the wood, grass, industrial crops, leaf, seed, fruit, and bast of plants are abundant in resources [4]. Composites made from natural fibers have some distinct characteristics compared to composites made from synthetic fibers. Composites based on natural fibers often have a lower specific weight than composites based on synthetic fibers. This can be advantageous in applications where lightness is essential. Although the density of natural fiber is low, it has a high specific strength and specific modulus. In composites, they can effectively transfer stress and enhance the mechanical properties of the matrix. For example, in specific application scenarios, the tensile strength and bending modulus of flax fiber-reinforced composites can be comparable to some glass fiber-reinforced composites, which is suitable for aerospace, automotive, and other fields where lightweight materials and high strength requirements coexist [5,6]. From a cost point of view, composites based on natural fibers generally have a lower cost than composites based on synthetic fibers. Natural fibers are often cheaper to produce and process, making them more economical for certain applications [7]. In terms of environmental impact, composites based on natural fibers are often considered more environmentally friendly than composites based on synthetic fibers due to their renewable origin and their faster degradation at the end of their life. From the renewable use of resources to the biodegradation of waste, natural fiber-reinforced composites run through the concept of circular economy. Its development contributes to the construction of a circular economy system with efficient use of resources and minimal waste, which is in line with the overall direction of global sustainable development. Developing natural fibers for potential applications in the pre-preg market is uniquely advantageous and worthy of attention.

To meet molding requirements, such as filament winding and 3D weaving, a pre-preg usually is in the form of a continuous tape or sheet. Unfortunately, unlike continuous synthetic fibers, natural fibers are limited in length. To make natural fibers continuous, twisting is the most often used method [8]. Other methods include preparing continuous hybrid yarns with thermoplastic fibers by various techniques such as blending [9], weaving [10], and wrapping spinning [11]. However, the twisted structure limits the penetration of the viscous thermoplastic melt into the core of rope and leaves voids in the pre-preg [12]. Secondly, the preparation process of blended yarn also requires the high spinnability of short natural fibers [13]. Third, the round cross-section of the twisted structure can not provide a flat surface for winding. Discontinuous natural fibers can also be connected in the form of bundles depending on the repeated melt and hardened characteristics of thermoplastic. Compared to the twisting structure, the fiber bundles are easier to impregnate. In a previous study, a high-density polyethylene sheet was used to impregnate straightened long sisal fibers (length ˃ 400 mm) and then connect them into a continuous pre-preg tape by hot pressing–cooling [4].

However, this method has great limitations for most short natural fibers such as the commonly used commercial short jute fiber bundles (the length is mostly between 5 and 38 mm) [14,15,16]. The flow of molten plastics severely affects the orientation and uniformity of the short fiber bundles; thus, it is unclear whether short fiber bundles can provide pre-pregs with higher tensile properties than continuous jute twine. A simple and effective method for preparing continuous pre-preg tapes directly from short natural fiber bundles remains to be developed.

Flexibility is an important requirement for pre-preg tapes used for filament winding, especially for winding small diameter pipes. In commonly used thermoplastics, linear low-density polyethylene (LLDPE) offers many performance advantages, such as flexibility, low melting point, and high weather resistance and tear-resistance. They are widely used as mulching and greenhouse film in agriculture. Every year, approximately more than 5 million tons of waste LLDPE films are discarded in the world [17,18], resulting in severe environmental pollution. Thus, recycled LLDPE is a better choice for preparing jute fiber pre-pregs, in terms of both resource utilization and property enhancement. At the same time, the hydrophilic behavior of natural fibers itself may lead to insufficient long-term durability of composites. Therefore, the matching of thermoplastic resin should be with a hydrophobic type. Guijun Xian’s study showed that the maximum water absorption and diffusion coefficient of fiber-reinforced polypropylene composites were lower than epoxy- and polyurethane-based composites [19]. Polyethylene resin has been widely used as a raw material for the preparation of water pipelines and has good water resistance [20].

In this study, commercial short jute fiber bundles were explored to reinforce LLDPE film to prepare continuous pre-preg tapes. Instead of mixing with plastic pellets and alternately layered spreading, jute fiber bundles were first spread parallel to each other on an LLDPE film and then rolled up to form a pre-roll. The roll enclosing fiber bundles was hot-pressed in a designed mold to form a pre-preg tape, where the fiber bundles were more parallel to the tape than the fibers in twine. In addition, when the fibers were rolled up with the film, the unevenness of spreading can be compensated by layers of overlapping, thus ensuring the uniformity of the pre-preg. This is the first time the feasibility of using the untwisted jute fiber bundle pre-preg tapes in filament winding applications has been evaluated. The interlayer fusion quality, surface flatness, ring tensile strength, and stiffness of their winding specimens were assessed. The purpose was to develop a new method to prepare continuous pre-pregs from discontinuous short natural fibers. If successful, many other types of short natural fibers, such as bamboo fibers, can also be directly used to produce continuous pre-preg tape with high performance.

## 2. Materials and Methods

### 2.1. Materials

**Figure 1:**
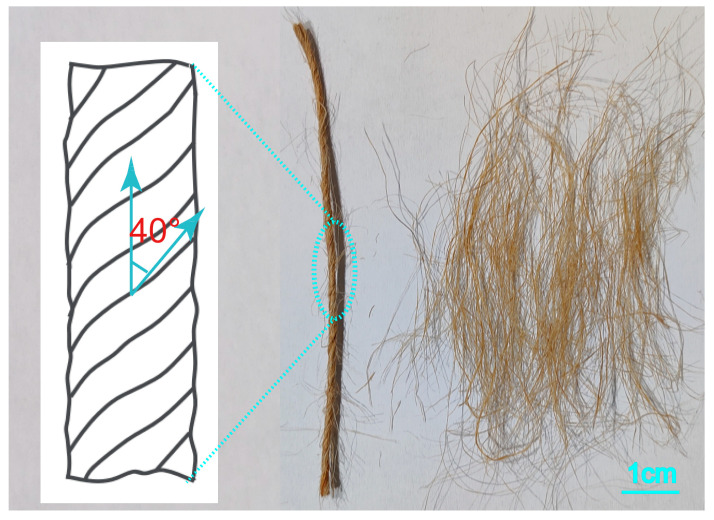
Twisted jute twine and carded short fiber bundle.

**Figure 2:**
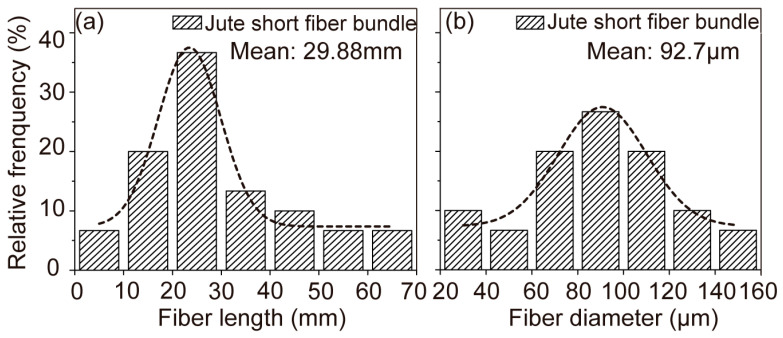
Frequency distribution of jute short fiber: (**a**) length and (**b**) diameter in bundles.

LLDPE films (tensile strength of 6.5 MPa, thickness of 18 μm, density of 0.92 g cm^−3,^ and melt flow index of 2.2 g (10 min)^−1^ were purchased from Xuzhou Heng Sheng Agricultural Film Co., Ltd. (Xuzhou, China). Jute twines and carded short fiber bundles were provided by Fujian Agriculture and Forestry University (Fujian, China). The short fiber bundles and twisted fiber twines of the same mass are displayed in Figure 1. The jute twine contained only one strand, and the twisting angle between the fiber directions in the strand and the central axis of the twine was 40° (65–85 fibers in each cross-section of the jute twine). The frequency distribution of the length and diameter of the jute fiber bundles is shown in Figure 2, obtained by Image J 8.0 software analysis and calculation. The fiber bundle length ranged from 15 to 35 mm and the diameter ranged from 70 to 110 µm. The tensile breaking strength of the jute twine and jute short fiber bundle were 61.2 × 10^−3^ N/tex and 36.4 × 10^−3^ N/tex, respectively.

### 2.2. Fabrication of Jute Fiber Bundle/LLDPE and Jute Twine/LLDPE Pre-Pregs

**Figure 3:**
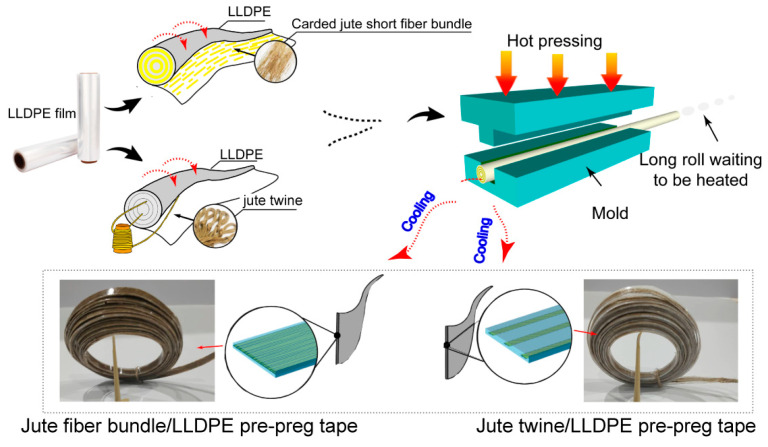
Preparation of jute fiber bundle/LLDPE and jute twine/LLDPE pre-preg tape.

The preparation schematic is shown in Figure 3. For the jute short fiber bundle/LLDPE pre-preg tape preparation, the short fibers were mechanically carded into well-oriented fiber bundles and oven-dried. Then, the fiber bundles were spread in parallel on a LLDPE film at a rate of 24–60 g/m^2^. In industry, this can be realized by mechanical forming, air forming, or a combination of the two. For example, the fiber spreading in the production of medium-density fiberboard and oriented particleboard. Afterward, fiber bundles were wrapped with the LLDPE film into a long enough roll. One end of the roll was put in a self-made mold (300 mm long, 10 mm wide, and 1 mm thick). This mold was pre-heated at 120 °C, 0 MPa for 2 min, hot-pressed at 120 °C, 10 MPa for 3 min, and finally cold-pressed at 10 MPa for 3 min to room temperature. According to the above parameters, the rest composite roll was hot-pressed one section by one section until they were all output as continuous pre-preg tapes. This process is easier to perform in the industrial continuous presses, which usually have heating and cooling stages. In this study, the overlap lengths of the fiber bundle ends in pre-preg were mostly in the range of 8–12 mm.

As a control, the jute twine/LLDPE pre-preg tape was prepared by the same process, as stated above. During this process, the thermoplastic LLDPE films fully played the role of carrier, glue, and matrix for the short fiber bundles. The mass fractions of jute fibers in pre-preg were set as 20%, 30%, 40%, and 50%. The details of all pre-preg samples are listed in Table 1. This manufacturing method can realize the continuous production of jute short fiber bundle/LLDPE pre-preg tapes.

## 3. Characterization

### 3.1. Void Content

The void content *V* (%) of the pre-preg was calculated as follows:

(1) $$V\%=\frac{\rho_{t}-\rho_{a}}{\rho_{t}}\times 100\%$$

(2) $$\rho_{t}=M_{f}\times \rho_{f}+(1-M_{f})\times \rho_{M}$$

where $\rho_{t}$ is the theoretical density of the pre-pregs calculated using Equation (2); $\rho_{a}$ is the actual density of the pre-pregs; $M_{f}$ is the fiber mass fraction; and $\rho_{f}$ and $\rho_{M}$ are the density of fiber and matrix, respectively.

### 3.2. Morphological Analysis

The distribution of jute fibers in pre-pregs was observed by the stereoscopic microscope (JSZ6, Changzhou Henglong Instrument Co., Ltd., Changzhou, China). The cross-sections of the pre-pregs were analyzed in a scanning electron microscope (SEM, FEI Quanta 200, FEI Co., Ltd., Hillsboro, TX, USA) at an acceleration voltage of 5 kV. Before the observation of SEM, the pre-preg specimens were fractured in liquid nitrogen for 2 min and then broken cryogenically. A small piece of a fractured pre-preg surface was placed over a metallic sample holder and subjected to gold sputtering. Some samples (jute twine/LLDPE) were obtained by cutting because the twisting structure of the rope was difficult to be fractured in liquid nitrogen.

### 3.3. Mechanical Testing

#### 3.3.1. Tensile Test for Jute Fiber Bundles and Twisted Jute Ropes

According to the standard NY T 2635-2014 (test method for the breaking tenacity of ramie fiber), jute fiber bundles and jute twines were equilibrated in a test environment with a temperature of 20 ± 2 °C and a humidity of 65% ± 2%. A 10 mm free length was chosen to enable suitable comparisons. About 0.2 g of parallel-aligned jute fiber bundles and jute twines were weighed to calculate their linear density $B_{\rho }$ by Equation (3):

(3) $$B_{\rho }=\frac{m}{L}\times 10^{3}$$

where $B_{\rho }$, *m*, and *L* are the linear density (tex), mass (g), and length (m) of jute fiber bundles and twisted jute twines, respectively. Then, the jute fiber bundles or jute twines were clamped on an electromechanical universal testing machine (CMT5504, MTS, Shenzhen, China) for tensile testing with a loading speed of 30 mm/min. At least ten specimens were tested for each group. The breaking strength of the fiber bundles or twines is expressed by *B_S_* (N/tex), which is calculated according to Equation (4).

(4) $$B_{S}=\frac{B_{F}}{B_{\rho }}$$

where *B_S_* and *B_F_* are the breaking strength (N/tex) and breaking load (N) of jute fiber bundles or jute twines, respectively.

#### 3.3.2. Tensile Testing on Pre-Pregs and Their Winding Specimens

**Figure 4:**
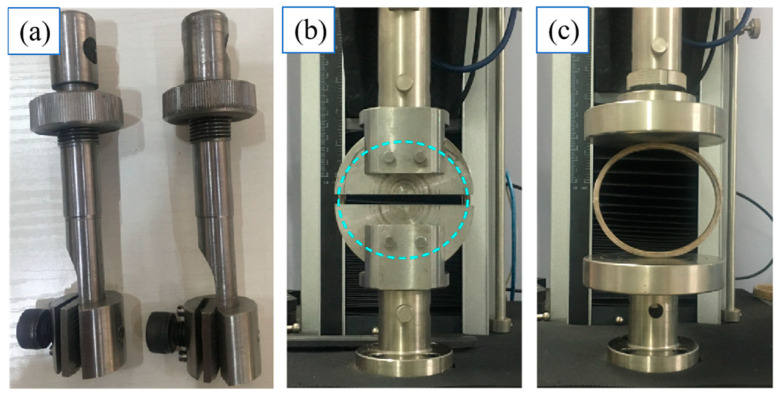
Fixture for longitudinal tensile testing (**a**), annular tensile testing (**b**), and ring stiffness testing (**c**) of the developed pre-preg.

According to ASTM D638 (Standard Test Method for Tensile Properties of Plastics), the longitudinal tensile properties of two types of pre-preg belts were determined using dumbbell-shaped samples (length of 115 mm, total width of 19 mm, narrow width of 6 mm, and thickness of 1 mm). The fixture used for the test (with a thread on the inside of the chuck to prevent slipping) is shown in Figure 4a.

In order to evaluate the potential usage of pre-pregs in winding, the annular tensile properties of pre-preg winding specimens were tested according to ASTM D 2290-2012 (Determination of Apparent Ring Tensile Strength of Plastic or Reinforced Plastic Pipe by Discrete Disk Method). The size of the test specimen was 76 mm nominal outer diameter × 10 mm width × 4.3 mm thickness, which was set according to GB/T 13663.1-2017 (Specification for Polyethylene Pipes for Water Supply). The test photo is shown in Figure 4b. The annular tensile strength *Ats* (MPa) of the specimen was calculated as follows:

(5) $$Ats=\frac{F}{2b\times d}$$

where *F*, *b,* and *d* are the maximum or pull off load (N), specimen width (mm), and thickness (mm), respectively. Both of the above tests were performed on an electromechanical universal testing machine with a load cell of 1000 N (CMT5504, MTS Systems Co., Ltd., Shenzhen, China). The loading speed is 2 mm/min. The strain at the fracture position of the ring specimen was measured by a mechanical extensometer [21]. At least five specimens were tested for each group. All tests were performed at 20 ± 2 °C and 65% ± 2% humidity.

#### 3.3.3. Ring Stiffness Testing on Pre-Preg Winding Specimens

According to ISO 9969: 2007 (Thermoplastic Pipe-Determination of Ring Stiffness), the ring stiffness test of the specimens (76 mm nominal outer diameter × 10 mm width × 4.3 mm thickness) obtained by winding the jute fiber bundle- or jute rope-reinforced LLDPE pre-preg tapes in the annular direction was carried out. The loading direction is shown in Figure 4c, and the loading speed was 2 mm/min. The temperature of the test environment was 20 ± 2 °C, the relative humidity was 65 ± 5%, and the test specimens were five in each group. The revised ring stiffness calculation formula is shown in (6):

(6) $$Rs=(0.0186+0.025\frac{y}{d_{i}})\frac{F}{Ly}\times 10^{6}$$

(7) $$\frac{y}{d_{i}}=0.03$$

where *Rs* is the ring stiffness (kN/m^2^), *F* is the load with respect to 3.0% deformation of the ring specimen (kN), *L* is the length of the ring specimen (mm), *y* is the amount of deformation relative to 3.0% of the ring specimen along the diameter (mm), and *d_i_* is the inner diameter of the ring specimen.

### 3.4. Statistical Analysis

All statistical calculations were completed by SPSS 19.0. A *p* < 0.05 was considered statistically significant.

## 4. Results and Discussion

### 4.1. Microstructure

#### 4.1.1. Planar Morphology of Jute Fiber Bundle/LLDPE and Twisted Jute Twine/LLDPE Pre-Pregs

**Figure 5:**
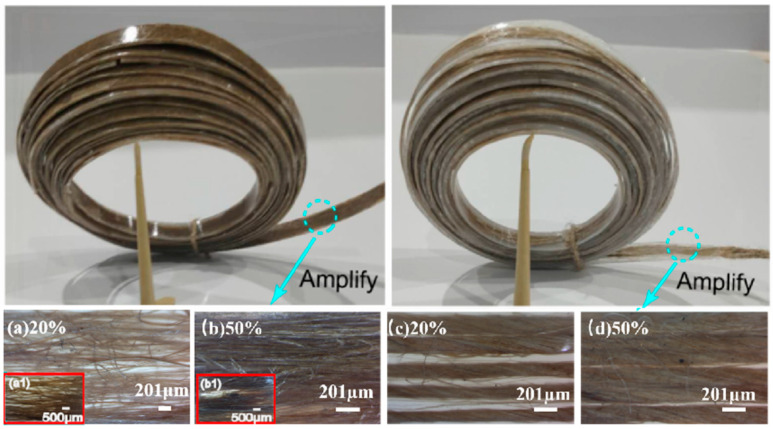
Microscopic observation of jute fiber distribution in continuous pre-preg tape: JFB/LLDPE pre-preg with jute mass fractions of 20% (**a**) and 50% (**b**); JT/LLDPE pre-preg with jute mass fractions of 20% (**c**) and 50% (**d**).

The orientation of the short fiber bundle is beneficial to obtain a better reinforcement effect [22]. Benefitting from the designed fabrication method, short fiber bundles were dispersed relatively parallel to each other in the JFB/LLDPE pre-preg (Figure 5a,b). When the fiber bundle or rope content increased from 20 to 50 wt.%, the gap between them became smaller, but the uniformity of the distribution became worse (Figure 5b,d), which increased the difficulty of LLDPE penetration. It is important to note that the fibers in the twine were arranged in a spiral shape along the twine axis at oblique angles. Fibers in the bundle were more loosely distributed compared to those in the twisted twine. These morphological differences would affect the performance of future pre-pregs.

#### 4.1.2. The Combination of Jute Fiber Bundle with LLDPE Matrix

**Figure 6:**
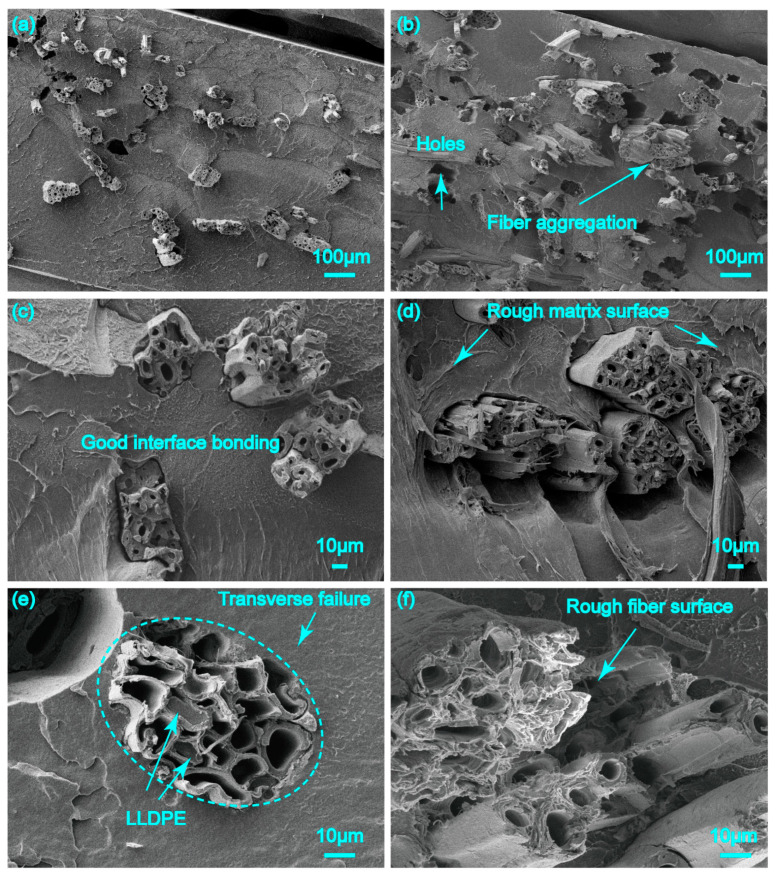
Cross-section of JFB/LLDPE pre-pregs under scanning electron microscopy with mass fractions of 20% (**a**,**c**,**e**) and 50% (**b**,**d**,**f**).

Figure 6a,b presents the cross-sectional images of the jute fiber bundle-reinforced LLDPE pre-preg at low multiples. When the bundles were present in low contents, they were randomly distributed throughout the cross-section. Observed at a higher magnification (Figure 6c), fibers were well covered by LLDPE at a lower bundle content, forming a good interface. Meanwhile, the fibers broke in a more brittle manner (Figure 6e), resulting in a transverse fracture. Some studies have shown that different fiber fracture modes reflect interfacial bonding, in which transverse cracks represent strong bonding regions [23]. Moreover, resin filling was found in the fractured fiber cavity, indicating that some short fiber bundles can be impregnated from the ends.

When the content of the jute fiber bundles increased to 50 wt.%, the larger bundle volume fraction was likely to cause fiber aggregation, resulting in a weak interface bond (Figure 6b,d).

When the content of the jute fiber bundles increased to 50 wt.%, the larger bundle volume fraction was likely to cause fiber aggregation, resulting in the poor wettability and bonding of resin to fiber. Under external loading, the fiber is easily pulled out from the resin matrix. In Figure 6b, there are many holes left after fiber fracture, suggesting the existence of a weak interface. Compared to Figure 6c for 20 wt.%, the introduction of more jute fibers rendered the fracture surface of both the polymer matrix and fiber rougher with more deformed microstructures (Figure 6d,f). In the jute bundles, most fibers were surrounded by the LLDPE matrix, even though aggregation had occurred (Figure 6d).

#### 4.1.3. The Combination of Twisted Jute Twine with LLDPE Matrix

**Figure 7:**
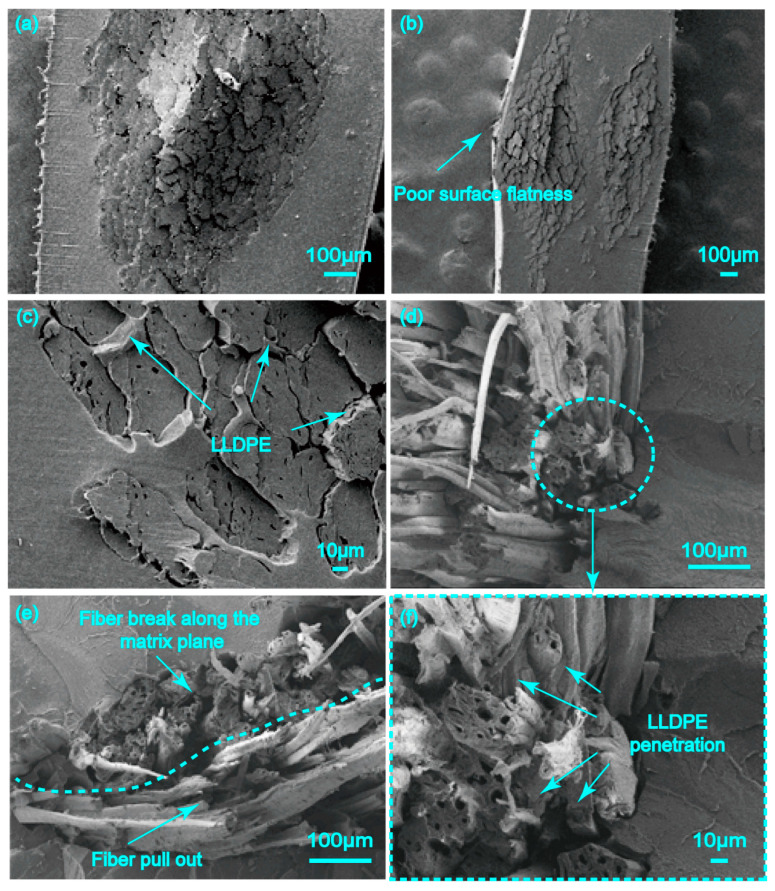
Fracture surface observation under scanning electron microscopy: (**a**,**c**,**e**) JT/LLDPE pre-pregs with mass fractions of 20%, (**b**,**d**,**f**) JT/LLDPE pre-pregs with mass fractions of 50%.

In Figure 7a,b, many voids exist among the fibers of twine, which indicated less LLDPE impregnation into the twisting fibers. This corresponds to the result shown in Table 1, indicating that the twisted twine-reinforced pre-preg had a larger void content than the short fiber bundle-reinforced pre-preg. By amplifying the different edge portions of the twine embedded in the matrix (Figure 7c), it was found that the penetration depth of the LLDPE in the twine was 100 to 180 µm, accounting for only about 17% to 30% of the twine radius.

For the fracture surface of the 20JR/LLDPE pre-preg (Figure 7e), the pulled-out twines contained fibers of different lengths, sticking out of the matrix. It can be observed that the fibers at the edge of the twine broke at the same plane as the matrix due to the strong penetration of the LLDPE matrix. However, the fibers inside the twine were extruded and separated from each other because of the lack of LLDPE matrix penetration. Furthermore, a significant loose bonding between the jute twine and LLDPE can be observed in Figure 7d,f.

Compared with short fiber bundles, the presence of twist prevents the thermoplastic matrix from penetrating into the fiber. This phenomena would decrease the reinforcement effect. In addition, compared to twisted twine (Figure 7a,b), fiber bundle presented the pre-preg flat surface (Figure 6a,b), which was important for composite molding.

### 4.2. Tensile Properties

#### 4.2.1. Tensile Breaking Strength of Jute Fiber Bundles and Twisted Jute Twines

**Figure 8:**
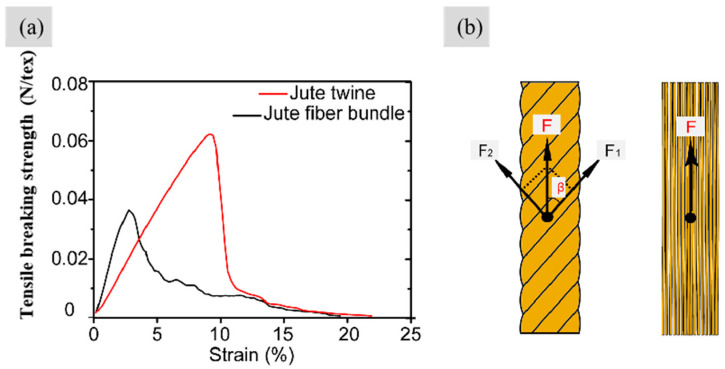
(**a**) Tensile breaking strength–strain curves and (**b**) force diagram of twisted jute twine and jute fiber bundle under tensile load.

A strength discussion of the two forms of reinforcement facilitates the subsequent interpretation of the tensile properties of the pre-preg (Figure 8a and Table 2). Table 2 shows that the jute twine had a much higher breaking strength than the fiber bundle. This is because the twist structure gives the outer layer fiber greater tension and centripetal force to bring the fibers into intimate contact. Moreover, some fibers interspersed between the inner and outer layers to produce entanglement, which increased frictional resistance and cohesion to some extent [24]. Figure 8b shows the force diagram of the fibers on the twisted jute twine under tensile loading, where *F*, *β*, *F*_1_, and *F*_2_ represent the forces that the fibers were subjected to along the twine axial direction (N), the angle between the fiber and the axis of the twine (°), the component force of F along the fiber direction (N), and the component force of *F* across the fiber (N), respectively. The actual tensile component (*F*_1_) acting in the fiber direction can be obtained as follows:

(8) $$F_{1}=F\times \cos\beta$$

Since 0 < *β* < 90°, *F*_1_ < *F*, the actual tensile force (*F*_1_) withstanding by fibers in jute twine was smaller than those in bundles. This increases the twine’s ability to withstand larger tensile forces. In addition, jute fiber bundles showed greater stiffness than twisted fiber twines (Figure 8a) because the misalignment of fibers in the twisted twine along the load direction provided less stiffness than oriented fiber bundles.

#### 4.2.2. Tensile Properties of the Pre-Pregs with Jute Fiber Bundle and Twisted Twine

**Figure 9:**
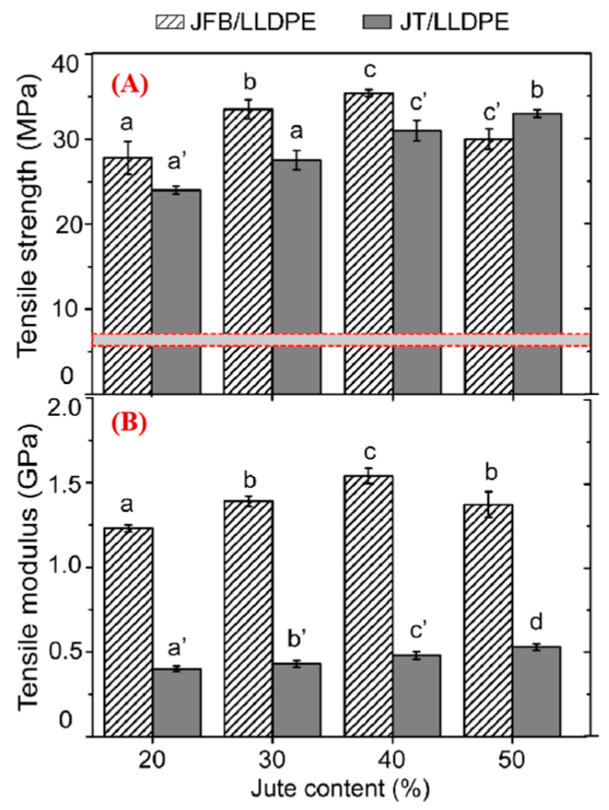
Longitudinal tensile properties of jute fiber bundle- and jute twine-reinforced pre-pregs: (**A**) tensile strength (red line represents the tensile strength of pure LLDPE), (**B**) tensile modulus. Note: When the same letter appears at the top of the column, there is no significant difference between any two groups. The letters mean the same in the following figures.

Though the fiber twine can withstand higher tensile breaking strength than bundles, Figure 9 shows a different trend when these fibers are embedded in the LLDPE matrix. When the jute mass fraction was less than 50%, JFB/LLDPE pre-preg presented greater longitudinal tensile strength than the JT/LLDPE pre-preg. The maximum tensile strength obtained for the JFB/LLDPE pre-preg with a fiber mass fraction of 40% was 32 MPa, 483% higher than that of the pure LLDPE and 16.7% higher than the JT/LLDPE pre-preg. The improvement in tensile strength leads to a considerable added value of discarded LLDPE films.

When the jute fiber content increased to 50%, the short fiber bundles did not further increase in tensile strength for the pre-preg. The fibers in the bundle were not completely bonded together at higher fiber levels, resulting in an interruption in force transmission.

In contrast, the jute twine with 50% content provided the pre-preg with a continuous increase in tensile strength compared to the 40% content. Despite the infiltration of LLDPE being inhibited when the short fibers were twisted into twines (Figure 7), the continuity, dispersibility, and high strength of the jute twine played positive roles in the stretching of the pre-preg. The spiraling fibers interlock with each other and thus exert a force advantage (Equation (8) and Figure 8).

In Figure 9B, the JFB/LLDPE pre-preg shows a much larger modulus than the JT/LLDPE pre-preg. This is attributed to a better fiber resin distribution in the JFB/LLDPE pre-preg than the JT/LLDPE pre-preg, leading to better load sharing between fibers. On the other hand, the misalignment of the fibers in the twisted twine can also result in a lower stiffness in the direction of load than the unidirectional fiber bundles. The significant advantage of fiber bundle-reinforced pre-pregs in terms of the elastic modulus would give the final product greater stiffness. When the fiber content increased to 50 wt%, the tensile modulus of JFB/LLDPE pre-preg showed a decreasing trend, which may be due to the fiber aggregation and interface bonding problems.

#### 4.2.3. Tensile Performance Evaluation of Developed Jute Pre-Pregs

The development of pre-pregs is an important step to optimize the reinforcing effect of natural fibers [25]. To evaluate the performance of these two types of pre-pregs (JFB/LLDPE and JT/LLDPE), the tensile properties of fiber-reinforced thermoplastic composites obtained from similar materials were summarized for comparison [26,27,28,29,30,31]. Table 3 shows that, in addition to the selection of fibers and matrix, the preparation method is one of the factors affecting the mechanical properties of composites. Compared to the extrusion and injection molding, compression molding gives the composite a higher tensile strength by maximally retaining the original length of the fibers. In addition, the dispersion and orientation of fibers are also important factors affecting the enhancement effect.

Compared with the natural fiber/thermoplastic composites listed in Table 3, the pre-pregs developed in this study are at a higher tensile strength level, owing to the good dispersion and orientation of jute short fibers. It is worth noting that the JFB/LLDPE and JT/LLDPE pre-pregs have reached comparable tensile strength to silane treated 30 wt% E-glass fiber/HDPE composites [23]. This makes it possible to broaden the application range of natural fibers and even replace synthetic fibers with natural fibers.

### 4.3. Filament Winding Demonstration of Developed Jute Pre-Preg Tapes

Except for the basic physical properties, the potential application of JFB/LLDPE and JT/LLDPE pre-preg tapes in filament winding was further exploited.

**Figure 10:**
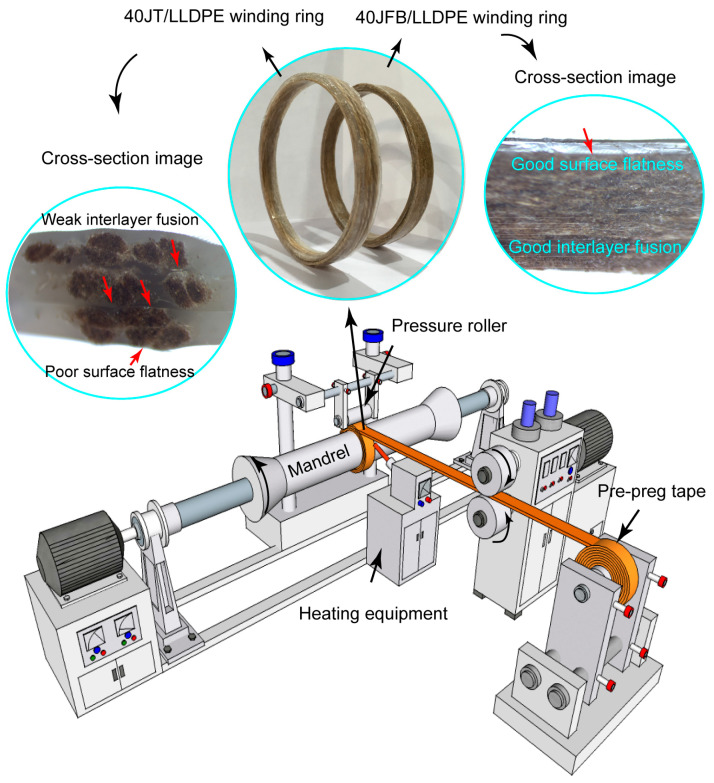
Filament winding process of continuous JFB/LLDPE and JT/LLDPE pre-preg tapes, as well as images of the obtained winding ring products (including enlarged cross-sectional morphology).

As shown in Figure 10, the pre-preg tape was heated and consolidated around a rotating mandrel with a circular geometric path, pressure, and tension to create a ring-shaped structural solid. The ring specimens were obtained by winding 40JFB/LLDPE and 40JT/LLDPE pre-preg tapes (see Figure 10). The cross-section of the fiber bundle ring exhibited good interlayer fusion, and no cracks or delamination were found. In contrast, the cross-section of the twine ring showed an unsatisfactory interlayer fusion state because of the poor LLDPE penetration into the core of the twisted twine.

Compared with the fiber bundle ring, the concentrated and stacked twines caused the 40JT/LLDPE winding ring to have an uneven surface structure, affecting the processing accuracy of the final product.

**Figure 11:**
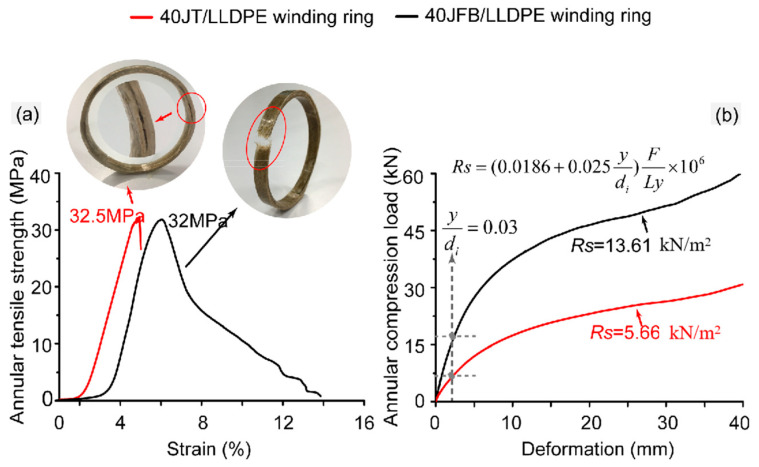
(**a**) Annular tensile stress–strain curves and (**b**) annular compression load–displacement curves of 40JFB/LLDPE and 40JT/LLDPE winding rings.

Furthermore, the annular tensile strength and ring stiffness are important parameters that characterize the performance of the tube against internal and external pressure, respectively [32]. Though there was no significant difference in annular tensile strength between jute twine ring (40JT/LLDPE) and fiber bundle ring (Figure 11a, Table 4), the former showed significantly smaller breaking elongation than the latter. After unloading, the fiber bundle ring showed a rough ductile fracture surface, accompanied by fiber breakage (Figure 11a). On the contrary, no obvious fracture behavior was found on the outside of the failed twine ring; however, the serious interlayer separation occurred inside (Figure 11a). It was suggested that the weak bonding area between twine pre-preg layers constituted the defect origin and stress concentration point, thus speeding up the structural failure under small deformation. The short-term fracture strain of the twine ring is often not easy to be detected and remedied in time, which threatens the stability of the overall structure.

In terms of ring stiffness (annular compression strength) (Figure 11b and Table 4), the fiber bundle ring showed an absolute advantage, with a value of 13.61 kN/m^2^, exceeding the 140.52% range of the fiber twine ring. These results demonstrate that the continuous pre-preg tape reinforced by short jute fiber bundles has the potential to be applied to filament winding and can exhibit a better forming performance.

**Figure 12:**
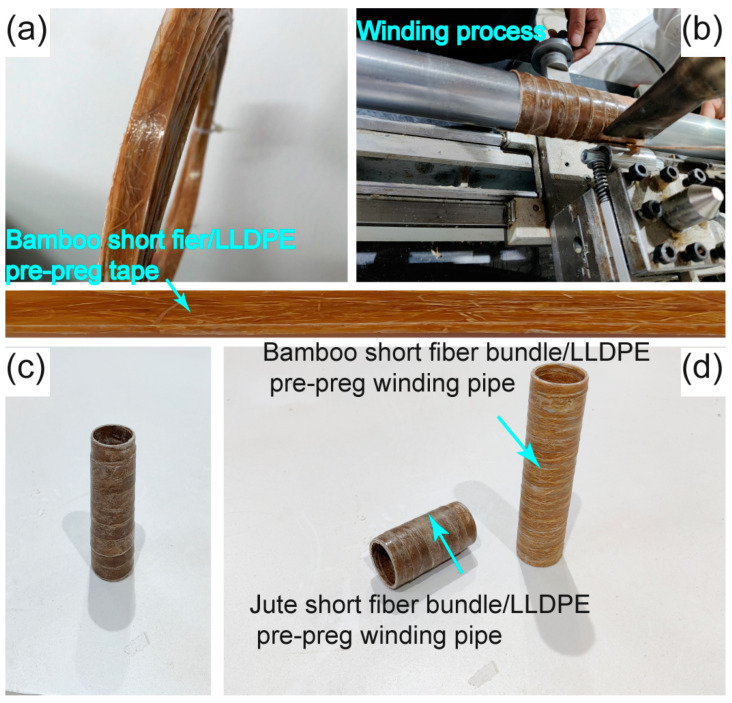
(**a**) Bamboo short fiber bundle/LLDPE pre-preg tape, (**b**–**d**) pictures of small-diameter pipes (outer diameters: 40 mm) wound by 40 wt% jute short fiber bundle/LLDPE pre-preg tape and 40 wt% bamboo short fiber bundle/LLDPE pre-preg tape.

Based on the method developed in this study, other types of short fiber-reinforced pre-preg tape can also be prepared and used in winding (Figure 12a). Figure 12b–d shows images of pipes (outer diameter: 40 mm) obtained by winding 40 wt.% jute short fiber bundle/LLDPE pre-preg tape and 40 wt.% bamboo short fiber bundle/LLDPE pre-preg tape, respectively. Because of the unique advantages of natural fibers such as greenness, light weight, insulation, and heat preservation, these pre-preg tape winding products are expected to be used in thermal insulation duct, cable protection pipe, medical antibacterial pipe, and other aspects.

## 5. Conclusions

In this study, short jute fiber bundles without twisting were used to prepare LLDPE pre-preg tapes. The short fiber bundles achieved unidirectional, uniform spreading and good impregnation in the continuous pre-preg tape. Though the untwisting structure exhibited a lower tensile strength for the fiber bundle, it could be impregnated well by LLDPE, resulting in a pre-preg with a higher longitudinal tensile strength and modulus compared to twisting structures when the jute mass fraction was between 20 and 40 wt.%. The development of short jute fiber bundle/LLDPE pre-preg also provides a potential application method for realizing the efficient utilization of waste LLDPE films. The tensile performance of the resulting jute/LLDPE pre-preg is equivalent to HDPE-based composite.

The experiment results of filament winding showed that the jute fiber bundle winding ring exhibited advantages in interlayer fusion quality, surface flatness, and ring stiffness. The poor interlayer fusion of the twine winding ring due to the poor LLDPE penetration is not conducive to the stability of the overall structure.

This research realized the continuous production of pre-preg tape reinforced with short jute fiber bundles. Many other kinds of short natural fibers, such as bamboo fibers, flax, hemp, etc., can also be used for the production of continuous pre-preg tape based on this method.

## Figures and Tables

**Table 1:** Labels of jute fiber bundle- and jute twine-reinforced pre-preg samples.

Pre-Preg	Ratio of Jute to LLDPE	Label	Void Content (%)
Jute fiber bundle/LLDPE	20:80	20JFB/LLDPE	3.80
	30:70	30JFB/LLDPE	4.31
	40:60	40JFB/LLDPE	5.04
	50:50	50JFB/LLDPE	6.22
Jute twine/LLDPE	20:80	20JT/LLDPE	5.80
	30:70	30JT/LLDPE	6.40
	40:60	40JT/LLDPE	7.55
	50:50	50JT/LLDPE	8.80

**Table 2:** Breaking strength (B_S_) calculation results of jute fiber bundle and jute twine.

Samples	Linear Density B_ρ_ (tex)	Breaking Load B_F_ (N)	Breaking Strength B_S_ (N/tex)
Jute twine	2.3 × 10^3^	140.77	61.2 × 10^−3^
Jute fiber bundle	1.9 × 10^3^	69.16	36.4 × 10^−3^

**Table 3:** Tensile properties of some of the natural fiber-reinforced thermoplastic composites.

Constituents of the Composites	Forming Method	Tensile Strength of Matrix (MPa)	Tensile Strength of Composites (MPa)	Tensile Modulus of Compisites (GPa)	Reference
30 wt.% Jute fiber/LLDPE	compression molding	-	32	0.82	(Niloy Rahaman et al., 2019) [27]
30 wt.% jute fiber/35 wt.%LDPE/35 wt.%LLDPE	melt mixing + compression molding	20.3 ± 0.7	16	0.53	(Choudhury and Adhikari, 2007) [28]
20 wt.% fique fiber mats/LLDPE	compression molding	15	17.6	1.37	(Hidalgo-Salazar and Juan, 2018) [29]
30 wt.%/hemp fiber/HDPE	melt mixing + compression molding	22	33	-	(Facca et al., 2007) [26]
30 wt.% hemp fiber/Poly(ester amide)	Injection molding	7	19	2	(Muthuraj et al., 2015) [30]
30 wt.% jute fiber/PP	Injection molding	28.5	27.5	1.75	(Kabir et al., 2010) [31]
30 wt.% silane treated E-glass fiber/HDPE	melt mixing + compression molding	22	34	-	(Facca et al., 2007) [26]

**Table 4:** Annular tensile strength (*Ats*) and ring stiffness (*Rs*) of pre-preg wound specimens (the same letter means no significant difference).

Pre-Preg Wound Specimen	*Ats* (MPa)	*Rs* (kN/m^2^)
40JFB/LLDPE	32 ± 0.92 ^a^	13.61 ± 0.31 ^c^
40JT/LLDPE	32.5 ± 1.43 ^a^	5.66 ± 0.13 ^b^

## Data Availability

The original contributions presented in this study are included in the article. Further inquiries can be directed to the corresponding author.
